# Artificial Intelligence, eHealth, and Telemedicine in Precision Medicine: From Digital Innovation to Clinical Integration

**DOI:** 10.3390/jpm16090484

**Published:** 2026-09-20

**Authors:** Mirella Veras

**Affiliations:** 1 Department of Physical Therapy, University of Manitoba, Winnipeg, MB R3E 0T6, Canada; mirella.veras@umanitoba.ca; 2 Centre on Aging, University of Manitoba, Winnipeg, MB R3E 0T6, Canada

## 1. Introduction: The Evolving Landscape of Digital Precision Medicine

Precision medicine has traditionally been associated with using genomic, molecular, and clinical information to tailor prevention, diagnosis, and treatment to individual patients [1]. More recently, however, the concept has expanded beyond biological characterization to include the growing availability of digital technologies that capture functional, behavioral, environmental, and patient-generated data [2,3]. Artificial intelligence (AI), eHealth, telemedicine, remote monitoring, wearable technologies, and immersive environments are increasingly creating opportunities to deliver care that is not only personalized, but also more continuous, adaptive, and responsive to changes in an individual’s needs [2,4].

This evolution is particularly relevant as healthcare systems face increasing demands related to population aging, chronic disease, mental health needs, workforce limitations [5], and inequitable access to specialized services [6]. Digital technologies may help extend care beyond traditional clinical environments, support earlier identification of health concerns, facilitate remote assessment and intervention, and enable treatment intensity or content to be adapted according to individual characteristics and progress [2,6]. At the same time, the rapid development of these technologies raises important questions regarding clinical validity, accessibility, privacy, equity, usability, governance, and integration into existing models of care [6,7].

The contributions (Fabio et al., 2025; Mansoor & Ansari, 2025; Mansoor & Ansari, 2024; Moevus et al., 2026; Veras et al., 2025) to the Special Issue, “eHealth, Telemedicine, and AI in the Precision Medicine Era,” illustrate this transition from digital innovation toward more personalized and clinically meaningful applications. Collectively, the published studies span remote rehabilitation for individuals with complex neurological conditions, AI-supported evaluation and prediction in mental health, extended reality (XR) for neuromusculoskeletal rehabilitation, and the systematic mapping of AI-powered technologies designed to support mobility and function in older adults. Rather than representing isolated technological developments, these contributions (Fabio et al., 2025; Mansoor & Ansari, 2025; Mansoor & Ansari, 2024; Moevus et al., 2026; Veras et al., 2025) highlight a broader shift toward digital models of care in which access, personalization, prediction, adaptation, and patient engagement are increasingly interconnected.

The studies also show that technological capability alone is insufficient to achieve meaningful precision care. Successful implementation requires aligning technologies with clinical goals, making them responsive to individual abilities and preferences, designing them with end users, and evaluating them within the social and healthcare contexts in which they will be used. This Special Issue therefore provides an opportunity not only to reflect on current advances in eHealth, telemedicine, and AI, but also to consider the scientific, ethical, and implementation challenges that must be addressed as these technologies move from innovation toward sustained clinical integration.

## 2. From Digital Innovation to Personalized Care

One of the clearest themes emerging from this Special Issue is the shift from using digital technologies primarily as alternative delivery mechanisms to using them as tools for increasingly personalized care. Telemedicine and telerehabilitation initially gained prominence largely because they could overcome geographic and logistical barriers. Their future contribution to personalized care, however, may depend on their capacity to respond to differences in diagnosis, functional status, environment, preferences, and treatment response.

This transition is illustrated by one study examining a structured telerehabilitation intervention for young girls with Rett syndrome. The remotely delivered program targeted foundational adaptive skills, including eye contact, object tracking, functional gestures, social engagement, and responsiveness to visual stimuli. The intervention was feasible and well tolerated, with preliminary improvements observed across several domains. Although larger controlled studies are required to establish effectiveness, the work demonstrates how remote rehabilitation can be adapted to the needs of individuals with complex neurological and communication impairments. Importantly, it also expands the role of telemedicine beyond convenience or access, positioning remote care as a potential platform for individualized rehabilitation.

The two contributions (Mansoor & Ansari, 2025; Mansoor & Ansari, 2024) addressing youth mental health further illustrate the potential of AI to support personalization at the level of both service delivery and early identification. Analysis of national telehealth data showed that the effectiveness and utilization of remote mental health services varied according to factors such as age, session frequency, previous diagnosis, socioeconomic context, and home environment. These findings suggest that telehealth outcomes cannot be understood solely by whether care is delivered remotely or in person. Individual and contextual characteristics may substantially influence who benefits from digital care and under what circumstances.

A complementary study used AI-powered analysis of social media data to identify patterns associated with emerging mental health concerns across multiple languages and digital platforms. The study demonstrates the potential of AI to identify behavioral and linguistic signals that may precede conventional recognition of clinical deterioration. Such approaches illustrate a broader change in precision medicine: the possibility of moving from episodic assessment toward more continuous and context-sensitive identification of changes in health status. At the same time, using digital behavioral data raises significant ethical considerations, including privacy, consent, cultural validity, potential stigmatization, and the need for appropriate human oversight.

Personalization also emerges strongly in the contribution focused on XR-based serious games for neuromusculoskeletal rehabilitation. The authors argue that clinically relevant XR systems should allow clinicians to adjust task difficulty, positioning, movement requirements, feedback, and other game parameters to match individual patients’ abilities and therapeutic goals. Their recommendations emphasize that personalization should not be limited to automated adaptation. Clinician input remains critical for refining therapeutic challenge, preventing compensatory movement strategies, monitoring progress, and ensuring that immersive technologies remain aligned with rehabilitation objectives. The proposed integration of AI offers an additional layer of adaptability, with the potential to modify difficulty and exercise selection dynamically using real-time patient performance data.

Finally, the protocol for an evidence and gap map of AI-powered technologies for mobility and function in older adults extends the discussion of personalization to the evidence base itself. The proposed map will examine not only which AI technologies are being used, but also how evidence is distributed across areas such as mobility, function, remote monitoring, health-service integration, interoperability, governance, humanization, and equity. The research process incorporates patient involvement, with particular attention to populations that may face barriers to digital healthcare, including rural, Indigenous, and other underserved communities [8].

Taken together, these contributions (Fabio et al., 2025; Mansoor & Ansari, 2025; Mansoor & Ansari, 2024; Moevus et al., 2026; Veras et al., 2025) suggest that technology-enabled personalized care should not be defined simply by the use of sophisticated technologies. Personalization arises from the interaction between technology, clinical expertise, patient characteristics, functional goals, environmental context, and patient preferences. The challenge for the next phase of digitally enabled care will therefore be to develop systems that adapt not only to clinical data, but also to the lived realities of the individuals and communities they are intended to serve.

## 3. Knowledge Gaps and Barriers to Clinical Integration

Despite rapid advances in artificial intelligence, telemedicine, and other digital health technologies, the contributions (Fabio et al., 2025; Mansoor & Ansari, 2025; Mansoor & Ansari, 2024; Moevus et al., 2026; Veras et al., 2025) to this Special Issue also underscore a persistent gap between technological innovation and sustained clinical integration. Demonstrating feasibility, predictive performance, or user engagement represents an important first step, but these outcomes alone do not establish clinical effectiveness, long-term benefit, scalability, or successful implementation across diverse healthcare settings. Moving digital innovations into routine practice therefore requires evidence beyond technical performance, including clinical outcomes, usability, accessibility, acceptability, safety, and organizational fit.

The telerehabilitation study involving young girls with Rett syndrome illustrates this challenge particularly well (Fabio et al., 2025). Although the intervention was feasible, well tolerated, and associated with preliminary improvements in adaptive skills, the authors emphasized the need for larger controlled studies to establish effectiveness. This reflects a broader issue in digital rehabilitation research, where promising early findings often emerge in small or highly selected populations before evidence is available on long-term outcomes, comparative effectiveness, or transferability across clinical and home environments.

Similar questions arise in AI-enabled mental health applications. The analysis of youth telehealth services demonstrated that outcomes varied according to individual and contextual factors, including age, previous diagnosis, socioeconomic characteristics, and home environment (Mansoor & Ansari, 2025). These findings highlight the importance of evaluating not only whether a digital intervention is effective on average, but also for whom, under what circumstances, and within which service models it is most likely to produce benefit. Such heterogeneity is central to personalized medicine, but it also creates challenges for generalizability and implementation.

The use of AI to identify emerging mental health concerns from social media data raises an additional set of questions concerning the translation of predictive performance into clinically meaningful action. High-performing algorithms do not automatically translate into improved patient outcomes (Mansoor & Ansari, 2024). Their clinical value depends on how clinicians interpret, communicate, and incorporate predictions into appropriate care pathways. False-positive and false-negative classifications, privacy concerns, informed consent, cultural differences, and the potential for stigmatization further emphasize the importance of human oversight and careful integration into existing clinical services.

Clinical integration also requires digital tools to align with professional workflows and therapeutic objectives. The XR rehabilitation contribution demonstrates that many emerging immersive technologies remain limited by insufficient customization options, inadequate data analytics, and poor alignment with established rehabilitation principles. (Moevus et al., 2026). Clinicians need to monitor performance, modify therapeutic parameters, provide feedback, and adapt interventions to patient progress. Technologies that cannot accommodate these requirements may remain technically innovative but clinically difficult to adopt. The authors’ emphasis on early stakeholder involvement reinforces the importance of co-design and iterative development in bridging this gap.

Implementation should also consider whether digital interventions operationalize person-centered care in a transparent and measurable way. A recent American Heart Association scientific statement examining stroke transitions of care found that person-centered components were often insufficiently described, making it difficult to determine how patient preferences, social and cultural context, family involvement, and continuity of care were incorporated into interventions [9]. The authors highlighted the need to delineate person-centered components explicitly, report relevant social and cultural characteristics, and clarify the role of family and nonmedical supports. These considerations are equally relevant to technology-enabled care, where implementation should extend beyond technical integration to ensure that technologies align with patients’ goals, preferences, cultural contexts, support systems, and transitions across care settings [9].

Interoperability and data governance represent additional barriers to implementation [7]. As digital platforms increasingly collect data through remote monitoring, mobile applications, wearable technologies, immersive systems, and AI-enabled tools, integrating these data into existing healthcare systems becomes increasingly important. Fragmented systems can limit continuity of care, duplicate clinician workload, and reduce the practical value of otherwise sophisticated technologies [7]. At the same time, collecting and exchanging health-related data requires robust approaches to privacy, security, transparency, and governance, particularly when AI systems rely on continuously generated or highly contextual personal information [6].

The evidence and gap map protocol focused on AI-powered technologies for mobility and function in older adults further highlights the need to assess digital health beyond effectiveness alone. The MERTH framework explicitly incorporates equity, health-service integration, interoperability, governance, and humanization, reflecting the multidimensional nature of implementation. These domains are particularly important because technologies that perform well under controlled conditions may not be accessible, acceptable, or feasible for the populations that could benefit from them most (Veras et al., 2025).

Together, the contributions (Fabio et al., 2025; Mansoor & Ansari, 2025; Mansoor & Ansari, 2024; Moevus et al., 2026; Veras et al., 2025) to this Special Issue suggest that the central challenge in technology-enabled precision care is no longer simply whether innovative technologies can be developed. The more consequential question is whether they can be translated into care that is clinically valid, adaptable, trusted, interoperable, and sustainable across real-world healthcare environments. Addressing this translational gap will require closer collaboration among patients, clinicians, researchers, developers, healthcare organizations, and policymakers throughout the technology lifecycle [8,9].

## 4. Equity, Human-Centered Design, and Responsible AI

As digital health technologies become increasingly embedded in healthcare, their potential benefits must be considered alongside questions of who can access them, whose needs they reflect, and how responsibly they are developed and implemented. Technologies designed to personalize care may still reproduce or deepen existing inequities if access to devices, connectivity, digital literacy, language, cultural relevance, disability-related accessibility, and socioeconomic context are not considered [8]. Equity must therefore be viewed not as an additional implementation outcome, but as a central dimension of responsible digital care.

The contributions (Fabio et al., 2025; Mansoor & Ansari, 2025; Mansoor & Ansari, 2024; Moevus et al., 2026; Veras et al., 2025) to this Special Issue illustrate several aspects of this challenge. In the analysis of telehealth services for youth mental health, socioeconomic circumstances and the home environment were associated with differences in utilization and outcomes. These findings reinforce that digital access alone does not guarantee equitable benefit. The effectiveness of telemedicine or telerehabilitation may be shaped by the conditions in which care is received, including access to technology, privacy within the home, digital literacy, family support, and the availability of complementary healthcare services. Consequently, expanding digital services without addressing the structural factors that influence their use may widen rather than reduce disparities [8].

Equity is also particularly relevant for populations that have historically experienced barriers to healthcare access, including older adults, people with disabilities, rural and remote populations, and Indigenous communities [8]. Equitable implementation also depends on healthcare professionals being prepared to recognize and respond to the social, cultural, linguistic, and socioeconomic factors that shape access to care and patient engagement. The evidence and gap map protocol included in this Special Issue explicitly incorporates equity, accessibility, health-service integration, interoperability, governance, and humanization as dimensions for evaluating AI-powered technologies designed to improve mobility and function in older adults. This broader perspective is important because the value of a digital intervention cannot be determined solely by its technological sophistication or effectiveness under controlled conditions. Its relevance also depends on whether the intended populations can access, understand, trust, and meaningfully use it.

Community-engaged research further suggests that priorities for digital health and AI may differ substantially across populations and settings [8]. Work conducted with First Nations communities in Manitoba, Canada has emphasized the importance of grounding digital health priorities in community-identified needs, accessibility, trust, cultural relevance, and local context [8]. Such considerations are especially important when technologies are intended for populations that have experienced historical and ongoing inequities within healthcare and research systems. Technology-enabled care should therefore avoid assuming that solutions developed in one context can be transferred unchanged to another.

Responsible AI adds another layer of complexity. AI systems may support prediction, personalization, monitoring, and clinical decision-making, but their use also raises concerns related to privacy, consent, transparency, bias, security, accountability, and appropriate human oversight [6,7]. These concerns are particularly evident in the study using AI-powered social media analysis to identify emerging mental health concerns. Although the findings demonstrated the potential of AI to detect clinically relevant behavioral patterns, the authors also highlighted challenges related to privacy, cultural validity, stigmatization, and the interpretation of algorithm-generated signals. In sensitive clinical contexts, AI outputs should therefore complement rather than replace professional judgment, with clearly defined responsibilities for how predictions are interpreted and acted upon.

Human-centered approaches can help address some of these challenges by ensuring that technologies are responsive to the abilities, preferences, and contexts of those who will use them. The XR rehabilitation contribution, for example, emphasizes the importance of clinician input, adaptable therapeutic parameters, usability, and early involvement of patients and professionals in technology development. Recent evidence across digital health similarly supports greater end-user involvement throughout the development and implementation process [10]. However, participatory and co-design approaches remain inconsistently applied and reported across the field. This represents an important area for future research rather than an already established feature of digital health innovation.

Ultimately, responsible digital care requires more than technically accurate algorithms or sophisticated platforms. It requires systems that are equitable in access, inclusive in development, transparent in operation, accountable in use, and flexible enough to reflect differences among individuals and communities. As AI, telemedicine, and eHealth technologies become more deeply integrated into care, these principles will be essential to ensuring that innovation translates into meaningful and equitable health benefits rather than creating new forms of digital exclusion.

## 5. Future Directions: Toward Adaptive and Integrated Digital Precision and Personalized Medicine

The next phase of technology-enabled personalized care will require a shift from isolated technological solutions toward adaptive, interoperable, and clinically integrated systems. The contributions (Fabio et al., 2025; Mansoor & Ansari, 2025; Mansoor & Ansari, 2024; Moevus et al., 2026; Veras et al., 2025) to this Special Issue demonstrate the potential of artificial intelligence, telemedicine, telerehabilitation, and immersive technologies to extend care, support personalization, and generate new forms of clinically relevant information. However, future progress will depend on whether these technologies can be evaluated beyond feasibility and technical performance and translated into sustainable models of care across diverse populations and healthcare environments.

A first priority is the generation of stronger real-world evidence. Many digital health technologies continue to be evaluated in small samples, selected populations, or early-stage feasibility studies. Future research should therefore include prospective multicenter studies, pragmatic trials, longitudinal follow-up, and implementation studies that assess not only clinical effectiveness but also adoption, adherence, safety, usability, scalability, and sustainability. This is particularly important for technologies intended to support chronic disease management, rehabilitation, and mental health, where benefits may depend on sustained engagement and integration into existing care pathways.

A second priority is the development of more adaptive and multimodal approaches to personalization. AI systems increasingly have the capacity to combine clinical, functional, behavioral, and patient-generated data, creating opportunities to adjust interventions over time according to individual progress and context. In rehabilitation, for example, adaptive systems may modify task difficulty, feedback, or exercise selection using real-time performance data, while maintaining clinician oversight. Similar approaches may support more individualized digital health pathways, remote monitoring strategies, and early identification of changes in health status. The challenge will be to ensure that greater algorithmic adaptability is accompanied by transparency, clinical relevance, and clearly defined boundaries for automated decision-making.

Interoperability will also be essential. Digital tools that operate independently of electronic health records, clinical documentation systems, and established referral pathways may increase fragmentation rather than improve care. Future platforms should therefore be designed to facilitate appropriate data exchange, minimize duplication of clinician workload, and support continuity across settings. Standards for interoperability, data quality, security, and governance will be increasingly important as information from wearable devices, mobile applications, telemedicine and rehabilitation platforms, immersive technologies, and AI-enabled systems becomes part of routine care [7].

Hybrid models of care will also become increasingly important. The future of telemedicine and telerehabilitation is unlikely to involve a simple replacement of in-person services. Instead, digital technologies may be most effective when used to complement face-to-face care, allowing patients to move between remote and in-person services according to clinical need, preference, functional capacity, geography, and stage of recovery. Such models may provide greater flexibility while preserving the relational and clinical components of care that cannot always be replicated digitally.

Equity must remain central to this transition. Future studies should evaluate whether digital technologies are accessible and effective across differences in age, disability, language, geography, socioeconomic position, culture, and digital literacy. Greater representation of underserved populations in the development and validation of AI systems will be necessary to reduce the risk of algorithmic bias and digital exclusion. Research should also examine the infrastructure and support needed for successful implementation in rural, remote, Indigenous, and resource-constrained settings, rather than assuming that access to a digital intervention equals meaningful access to care.

Responsible AI will require continued attention to privacy, transparency, accountability, safety, and human oversight. As predictive and adaptive systems become more sophisticated, researchers and healthcare organizations will need to define how algorithmic outputs should inform clinical decisions, when professional review is required, and how uncertainty or error should be communicated. Particularly in sensitive domains such as mental health, AI should support clinical judgment rather than function as an autonomous substitute for it. Clear governance structures and ongoing monitoring will be essential as systems evolve after deployment.

Another important gap concerns the extent to which patients, clinicians, caregivers, and communities influence digital health technologies. Although several contributions (Fabio et al., 2025; Mansoor & Ansari, 2025; Mansoor & Ansari, 2024; Moevus et al., 2026; Veras et al., 2025) to this Special Issue emphasize the importance of user needs and contextual relevance, participatory and co-design approaches remain inconsistently integrated across the broader field. Future work should move beyond consultation toward more meaningful involvement of end users in defining priorities, selecting outcomes, developing interfaces, and evaluating implementation. This gap is especially relevant as digitally enabled care becomes more deeply embedded in care and will warrant greater attention in the next phase of research.

Finally, future evaluation should extend beyond whether a technology works to whether it delivers value. Economic evaluation, implementation costs, workforce implications, training requirements, reimbursement, sustainability, and health-system impact should increasingly accompany assessments of clinical effectiveness. Without this broader evidence base, even promising technologies may struggle to move from pilot projects into routine and equitable care.

This distinction between technological capability and human purpose is not entirely new. In Aristotelian terms, the value of a tool or practice is ultimately shaped by the end it serves [11]. Digital innovation should therefore remain a means of advancing human well-being, participation, and quality of life, rather than becoming an end in itself. Taken together, these priorities suggest that the future of technology-enabled precision care will depend less on developing increasingly complex technologies and more on the capacity to integrate them responsibly into healthcare systems. The most successful approaches will likely be those that are adaptive without being opaque, personalized without being exclusionary, digitally enabled without becoming disconnected from clinical care, and innovative without losing sight of the needs of the people and communities they are intended to serve.

## 6. Conclusions

The contributions (Fabio et al., 2025; Mansoor & Ansari, 2025; Mansoor & Ansari, 2024; Moevus et al., 2026; Veras et al., 2025) to this Special Issue illustrate the growing role of artificial intelligence, eHealth, telemedicine, telerehabilitation, and immersive technologies in advancing precision and personalized medicine. Across rehabilitation, mental health, healthy aging, and digital care delivery, the studies demonstrate important opportunities to expand access, support more individualized interventions, improve monitoring, and generate new forms of clinically relevant information.

At the same time, the evidence presented here reinforces that technological innovation alone is not sufficient. The future impact of technology-enabled personalized care will depend on whether these technologies can be clinically validated, integrated into existing care pathways, aligned with professional workflows, supported by appropriate governance, and implemented in ways that are equitable, accessible, and responsive to the needs of diverse populations. Human oversight, interoperability, transparency, sustainability, and attention to social and cultural context will remain central to responsible implementation.

Important gaps also remain in the meaningful involvement of patients, clinicians, caregivers, and communities throughout the development and implementation of digital health technologies. Participatory and co-design approaches are still inconsistently applied, and greater attention to these methods will be essential to ensure that future innovations are relevant, acceptable, and grounded in real-world priorities. This gap also provides a natural direction for the forthcoming second edition of the Special Issue, with greater emphasis on co-design, responsible innovation, and translating digital technologies into equitable and clinically meaningful care.

Ultimately, progress in digital precision medicine should be judged not by technologic sophistication alone, but by whether innovation improves care in ways that are clinically meaningful, equitable, sustainable, and responsive to the people and communities it is intended to serve.
